# Corneal nerve healing after *in situ* laser nerve transection

**DOI:** 10.1371/journal.pone.0218879

**Published:** 2019-06-27

**Authors:** Joy Sarkar, Behrad Milani, Eunjae Kim, Seungwon An, Jieun Kwon, Sandeep Jain

**Affiliations:** Department of Ophthalmology and Visual Sciences, University of Illinois at Chicago, College of Medicine, Chicago, IL, United States of America; Cedars-Sinai Medical Center, UNITED STATES

## Abstract

**Purpose:**

We have previously reported that lamellar dissection of the cornea transects stromal nerves, and that regenerating neurites form a dense net along the surgical plane. In these experiments, we have disrupted the stromal nerve trunks *in situ*, without incising the cornea, to determine the regeneration events in the absence of a surgical plane.

**Methods:**

*Thy1*-YFP mice were anesthetized and *in vivo* images of the corneal nerves were obtained with a wide-field stereofluorescent microscope. A far infrared XYRCOS Laser attached to 20X objective of an upright microscope was used to perform *in situ* transection of the stromal nerves. 3 types of laser transections were performed (n = 5/group): (i) point transection (a single cut); (ii) segmental transection (two cuts enclosing a segment of nerve trunk); and (iii) annular transection (cuts on all nerve trunks crossing the perimeter of a 0.8 mm diameter circular area centered on the corneal apex). Mice were imaged sequentially for 4 weeks thereafter to assess nerve degeneration (disappearance or weakening of original fluorescence intensity) or regeneration (appearance of new fluorescent fronds). Beta-3-tubulin immunostaining was performed on corneal whole-mounts to demonstrate nerve disruption.

**Results:**

The pattern of stromal nerves in corneas of the same mouse and in corneas of littermates was dissimilar. Two distinct patterns were observed, often within the same cornea: (i) interconnected trunks that spanned limbus to limbus; or (ii) dichotomously branching trunks that terminate at the corneal apex. Point transections did not cause degeneration of proximal or distal segment in interconnected trunks, but resulted in degeneration of distal segment of branching trunks. In segmental transections, the nerve segment enclosed within the two laser cuts degenerated. Lack of beta-3 tubulin staining at transection site confirmed nerve transection. In interconnected trunks, at 4 weeks, a hyperfluorescent plaque filled the gap created by the transection. In annular transections, some nerve trunks degenerated, while others regained or retained fluorescence.

**Conclusions:**

Interconnected stromal nerves in murine corneas do not degenerate after *in situ* point transection and show evidence of healing at the site of disruption. Presence or absence of a surgical plane influences corneal nerve regeneration after transection.

## Introduction

The cornea is the most densely innervated structure in the human body and the trigeminal ganglion provides sensory innervation to the cornea. Corneal nerves influence stimuli (touch, temperature and pain) perception and blink reflex, tear formation and maintenance of hydration as well as wound healing and avoidance of injury [1–7]. Ocular diseases such as Neurotrophic Keratitis and Dry Eye Disease cause considerable morbidity which is attributed to corneal nerve dysfunction [8–9]. Additionally, several studies have reported that routine surgical procedures in ophthalmic practice such as corneal transplantation, photorefractive keratectomy (PRK), radial keratotomy and laser-assisted *in situ* keratomileusis (LASIK) cause disruption and dysfunction of corneal nerves [10]. Corneal nerve regeneration spans several years after surgical transection and the nerve density never returns to pre-surgery levels [11, 12]. Although there has been a lot of progress in the field over the years, there is a paucity in our knowledge and understanding of the underlying mechanisms governing corneal nerve regeneration hence the reason for its high relevance in the ophthalmology field [13]. Prior studies from our lab using the *thy1*-YFP (yellow fluorescent protein) transgenic mouse model (in which the nerves fluoresce yellow) have shown that after lamellar surgery nerve regeneration occurred via sprouting at the proximal end of stromal trunks and these regenerated nerves sometimes do not demonstrate the same nerve pattern as observed before surgery [14]. We have also reported the infiltration of myeloid-derived YFP fluorescent cells in the cornea after nerve transecting surgery [14–16]. Although there are numerous studies on nerve loss, healing and regeneration after injury to the corneal nerves due to surgery, there is relatively scant knowledge on corneal nerve loss and regeneration in non-surgery scenarios of neurotrophic corneas wherein there is an absence of a surgical plane.

In our current study, we investigated the regeneration or healing of corneal nerves after an injury delivered directly and specifically to the corneal nerve trunks without creating any surgical planes. Such an injury was likely to somewhat replicate a disease of the nerve trunk without any surrounding anomaly. We proposed these investigations since our previous studies showed that corneal nerves regenerate along a surgical plane created by manual lamellar dissection of the cornea or an excimer laser annular keratectomy.

## Materials and methods

### Ethics statement

All animal experiments were conducted in strict accordance with the recommendations in the Guide for the Care and Use of Laboratory Animals of the National Institutes of Health. The animal protocol was approved by the Institutional Animal Care and Use Committee (IACUC) of the University of Illinois at Chicago (Protocol Number: 13–159). *Thy1*-YFP neurofluorescent homozygous adult mice (6–8 weeks old) were purchased from Jackson Laboratories (Bar Harbor, ME), and colonies were established by inbreeding. For *in vivo* experiments, mice were anesthetized with intraperitoneal injections of ketamine (20 mg/kg; Phoenix Scientific, St. Joseph, MO) and xylazine (6 mg/kg; Phoenix Scientific). For terminal experiments, mice were sacrificed according to the IACUC protocol. Euthanasia was performed by CO_2_ inhalation followed by cervical dislocation in adult animals. These procedures were chosen based on their reproducibility and the fact they cause no discomfort to the animals. These methods are consistent with the recommendations of the Panel on Euthanasia of the American Veterinary Medical Association. All efforts were made to minimize suffering.

### *In situ* laser transection

Experiments involving *in situ* transection of the mouse corneal stromal nerves were performed using a far-infrared XYRCOS Laser (Hamilton Thorne Inc; Beverly, MA) which permits non-contact ablation of targeted membranes or structures. The XYRCOS laser module consists of a high power, Class 1, 1460 nm infrared laser plus RED-i target locator integrated into a 20X objective and is compatible with most inverted microscopes. The XYRCOS laser attaches to the turret just like a typical objective and allows full use of all the microscopes standard features, such as fluorescence and Hoffman imaging. In addition, the laser is factory-aligned and locked in place to ensure safe ablation. In our setup the XYRCOS Laser was attached to a Zeiss AxioExaminer A1 Upright Microscope (Carl Zeiss Microscopy, Thornwood, NY). After initial baseline images (day 0) before surgery using a Zeiss Stereolumar microscope (details in next section), three types of nerve transection were performed using the XYRCOS laser (Pulse: 200 μs; Power: 100%); (i) single nerve cut in either an interconnected trunk or stromal nerve ending; (ii) segmental nerve injury on an interconnected stromal nerve trunk; and (iii) annular nerve transections. The mice were followed up after surgery and sequential stereomicroscopic images were taken over the next 4 weeks after nerve transections to assess changes in fluorescence intensity and pattern of regenerating nerve fronds, if any. Only stromal nerves were included in the analysis. Subbasal hairpin nerves were excluded.

### *In vivo* stereofluorescent microscopy

Initial baseline (Day 0) and serial imaging after nerve transection surgeries was performed using a fluorescence stereomicroscope (StereoLumar V.12, Carl Zeiss Microscopy, Thornwood, NY) equipped with a digital camera (Axiocam MRm) and software (AxioVision 4.0) as described previously [14]. An anesthetized mouse was placed on the stereoscope stage. Seven microliters of proparacaine (0.5%, Bausch & Lomb, Tampa, FL) was applied for 3 min, and the pupil was constricted with 0.01% Carbachol intraocular solution (Miostat, Alcon) for 5 min. Z-stack images were obtained at 5-μm intervals and compacted into one maximum intensity projection (MIP) image after alignment using Zeiss AxioVision software. Brightfield images were taken (S1 Fig) to confirm corneal transparency after nerve transection surgeries.

### Corneal whole-mount preparation and confocal microscopy

Mice were sacrificed and corneal whole mounts were prepared as described previously [14]. Corneas were excised and directly fixed in 4% paraformaldehyde (PFA) overnight at 4°C. The corneas were washed thrice with PBS for 5 min each at room temperature. This was followed by permeabilization of the corneas in PBS containing 3% NP40, 3% BSA, 0.25% gelatin, 5mM EDTA for 12 h at room temperature. The corneas were then washed thrice with PBS for 5 min each at room temperature. The corneas were then blocked in Blocking solution (PBS containing 0.025% NP40, 1% BSA and 2.5% Donkey Serum) overnight at 4°C. Furthermore, the corneas were incubated with primary antibody at appropriate dilution in blocking solution overnight at 4°C followed by 3 washes with PBS for 5 min each at room temperature. Fluorescence-coupled secondary antibodies in blocking solution at appropriate dilutions were then added to the corneas and incubated overnight at 4°C. The corneas were then washed with PBS thrice for 5 min each at room temperature. Corneas were mounted onto glass slides with a drop of 4', 6-diamidino-2-phenylindole (DAPI)-containing mounting medium and covered with a coverslip. Primary and secondary antibodies used were rabbit anti-beta III Tubulin (Abcam; ab18207; dilution 1:100) and donkey anti-rabbit Alexa Fluor 594 IgG (Abcam; ab150076; dilution 1:1000) respectively. To study the corneal nerve topography, we acquired confocal Z-stack images of corneal whole-mounts and performed 3D reconstruction using an LSM 710 confocal microscope (Carl Zeiss Meditec GmbH) and images were further processed with the Zeiss LSM Zen Imaging and Analysis Software.

### Statistical analyses

Statistical analyses were performed using Microsoft Excel. A Student’s t-test was used to compare mean values between groups. Results are shown as Mean ± SEM.

## Results

### Architecture and pattern of corneal nerves

**Fig 1:**
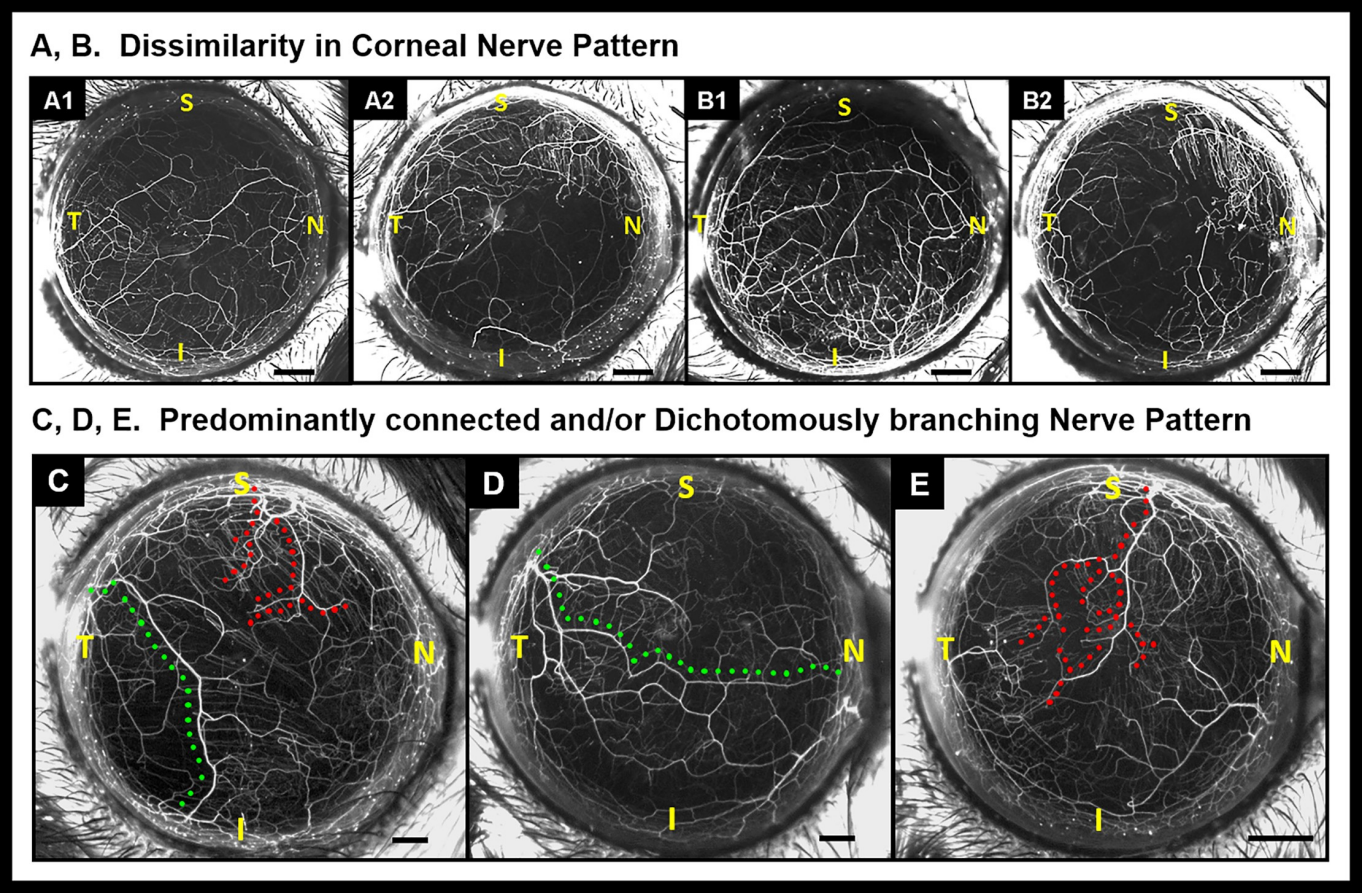
Dissimilarity in corneal nerve patterns. Stereoflourescent microscope image of cornea shows nerve patterns are dissimilar within the same mouse (OS and OD; A1 and A2 respectively) and between littermates (OS of two littermates; B1 and B2 respectively). (C—E) Stereoflourescent microscope images of corneal nerves showing different branching patterns. (C) Dichotomously branching trunks terminate near the corneal apex (red dotted line) and interconnected nerve trunks (green dotted line) span limbus to limbus. (D) Interconnected pattern (green dotted line). (E) Dichotomously branching pattern (red dotted line). S: Superior; I: Inferior; N: Nasal; T: Temporal position; Black scale bars in Panels A—E: 500 μm.

The branching pattern of corneal nerves shows considerable dissimilarity between the left eye (Fig 1. A1) and right eyes (Fig 1. A2) of the mice as well as between ipsilateral eyes of littermates (Fig 1. B1 and B2, left eye of littermates). Stromal nerve trunks enter the cornea at the limbus and show two different patterns: (i) Interconnected pattern with nerve trunks spanning limbus to limbus (Fig 1 Panel C and D, green dotted lines) or (ii) Dichotomous branching pattern with nerve trunks terminating at variable distances from the corneal apex (Fig 1 Panel C and E red dotted lines).

### Corneal nerve laser transection

**Fig 2:**
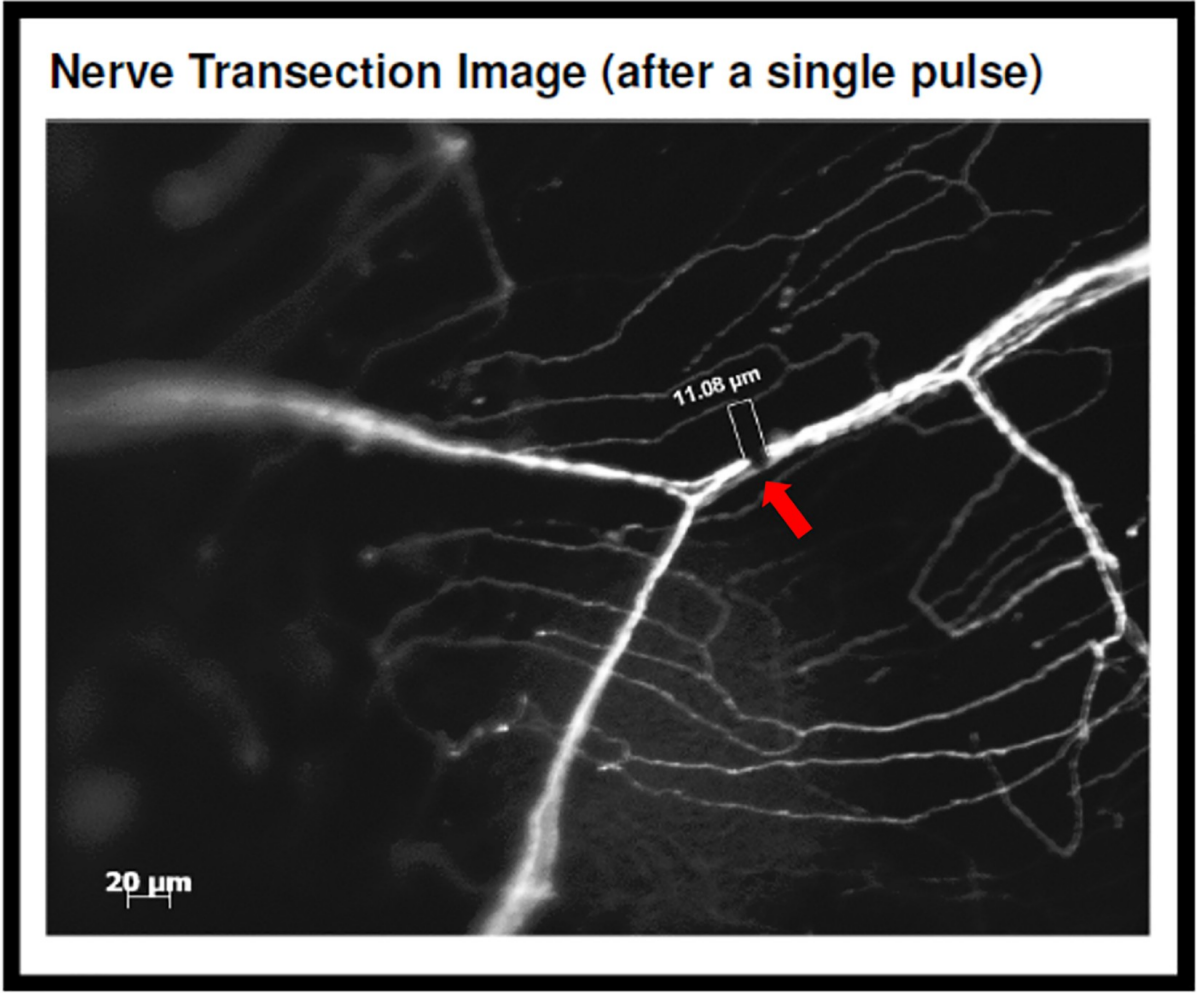
Fluorescent microscope image of *in situ* nerve transection. After a single laser pulse, loss of nerve trunk fluorescence is noticed in the ablated area. Red arrow shows location of laser ablation. White scale bar: 20 μm.

To characterize corneal nerve healing and regeneration in the absence of a surgical plane, *in situ* transection of the mouse corneal stromal nerves was performed using a far-infrared XYRCOS Laser (Hamilton Thorne Inc; Beverly, MA) which permitted non-contact ablation of targeted membrane. Nerve trunk fluorescence was lost in the ablated area (Fig 2, arrow). After a single pulse with the laser (pulse: 200 μs; power: 100%), the size of the transection zone was 11.33 ± 0.25 μm (Mean ± SEM).

**Fig 3:**
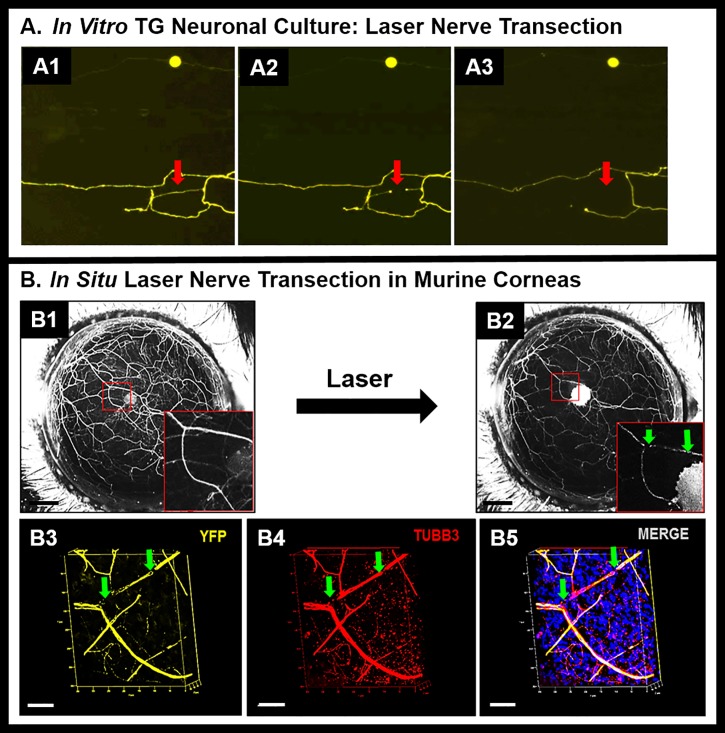
**(A) *In vitro* neurite transection**. Intact neurite (A1), transected neurite (A2), and disrupted neurite (A3) in TG neuronal cell culture. Red arrows show location of laser ablation. **(B) *In situ* nerve transection.** *In vivo* stereofluorescent images of corneal nerve before (B1) and after (B2) laser transection (inset: a dichotomously branching nerve trunk showing the ablation site that is marked with green arrows). (B3-B5) Confocal fluorescent microscope images of corneal whole mounts showing discontinuity in YFP fluorescence at the site of laser ablation (B3). Immunostaining for beta-III tubulin shows corresponding discontinuity in the nerve trunk (B4); thus confirming structural discontinuity in the nerve trunk (B5). White scale bars in Panels (A1- A3): 250 μm; Black scale bars in panels (B1-B2): 500 μm; White scale bars in Panels (B3 –B5): 50 μm.

To confirm that the laser pulse causes nerve ablation, we performed transection of neurites in *in vitro* trigeminal ganglion cell cultures (Fig 3A1–3A3). After a single laser pulse to a neurite (Fig 3. A1, arrow), a zone of loss of fluorescence was seen (A2, arrow), that progressed to disappearance of the whole neurite (Fig 3. A3, arrow). To further confirm that the laser pulse causes nerve ablation and not just loss of fluorescence, we performed immunohistochemistry of a nerve structural protein (beta-3-tubulin) after *in situ* nerve ablation. Absence of beta-3-tubulin staining in the zone of laser ablation confirms that the nerve trunk is discontinuous in the ablated area.

### Nerve healing after *in situ* nerve transection

Three types of laser nerve transections were performed in murine corneas using the XYRCOS laser; (I) Nerve transection at a single point along the trunk in either an interconnected nerve spanning limbus to limbus or dichotomously branching nerve that ends near the corneal apex; (II) Nerve transection at two points on an interconnected nerve; and (III) Annular nerve transections. Sequential stereomicroscopic images were taken over the next 8 weeks after nerve transections to assess changes in fluorescence intensity and pattern of regenerating nerve fronds.

### I. Single point transection of corneal nerves

**Fig 4:**
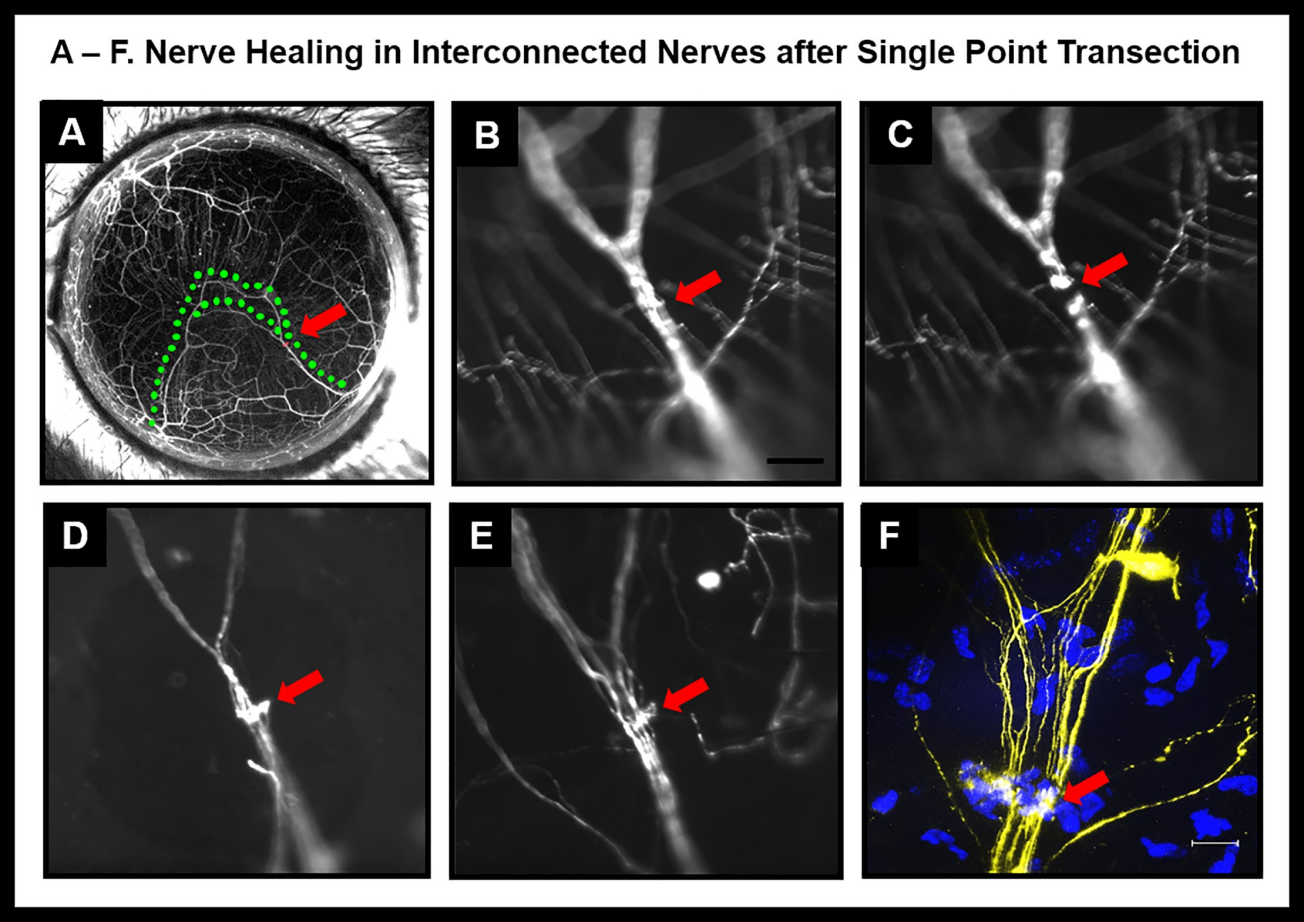
Nerve healing in interconnected nerves after single point transection. (A): Stereofluorescent image of cornea prior to nerve transection (red arrow indicates point of planned transection; green dotted lines mark the interconnected nerves. (B-E) Wide-field fluorescent image showing nerve prior to transection (B), immediately after transection (C), four weeks after transection (D) and six weeks after transection (E). (F) Confocal image of transected area.

**Fig 5:**
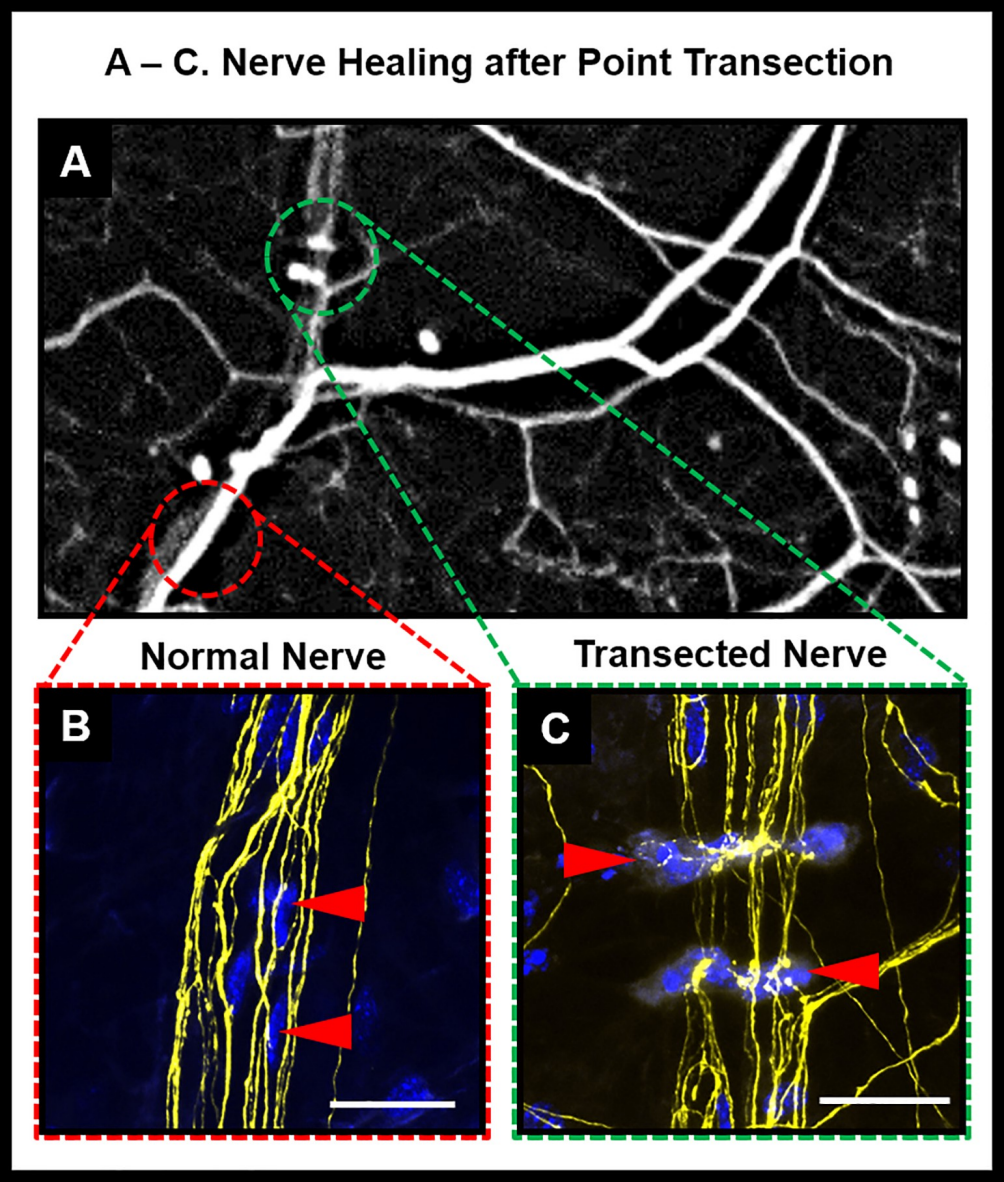
Nerve healing after point transection. (A): Magnified area of a stereofluorescent image of cornea two weeks after point transection (green dotted lines mark the transected area with cells; red dotted lines mark intact area of a normal untransected region along the nerve (B) inset shows magnified view of normal untransected nerve with cells oriented parallel along the nerve fibers (C) inset shows magnified view of transected area two weeks after ablation with cells oriented in perpendicular direction to the nerve.

**Fig 6:**
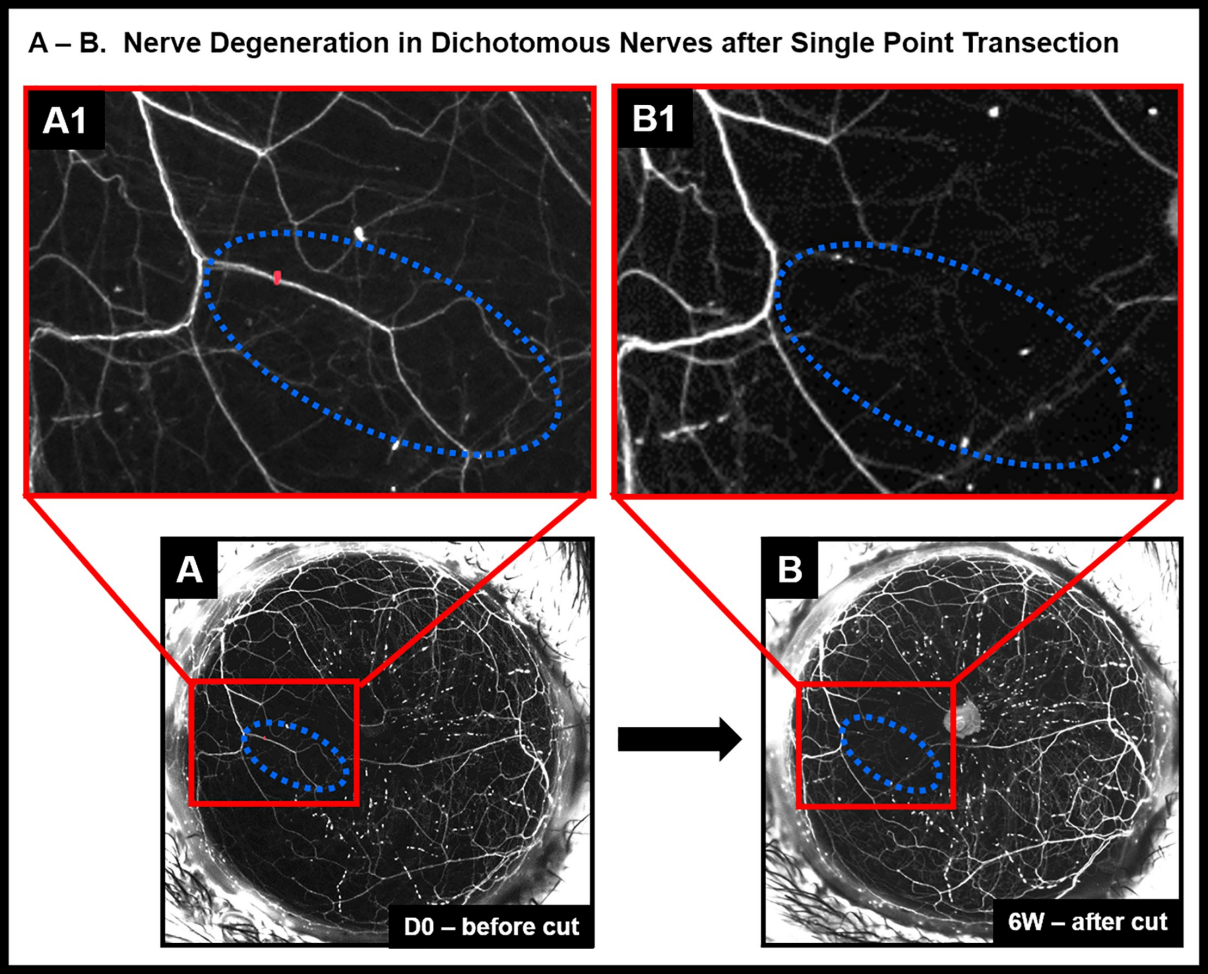
Nerve healing in dichotomous nerves after single point transection. Dichotomous nerves before transection (A) and six weeks after transection (B). Red mark indicates point of transection; blue dotted lines mark the zone around the transected dichotomous nerve. Insets show magnified view of the dichotomous nerve before transection (A1) and disappearance of the nerve fluorescence six weeks after transection (B1)., Panels A and B; Panels A1 and B1 are magnified insets of A and B respectively.

In interconnected nerves (Fig 4A, green dotted line) a single point ablation was performed. The continuous nerve trunk before ablation (Fig 4B) developed an area of discontinuity after laser ablation (Fig 4C, red arrow). The laser ablation did not cause degeneration of proximal or distal segment in interconnected trunks. By weeks 3 to 4, a hyperfluorescent plaque developed at the ablated nerve site (Fig 4D). By weeks 6 to 7, the nerve trunk appeared continuous but the area of injury remained hyperfluorescent (Fig 4E). Mice were sacrificed at week 8 and whole mount confocal imaging was performed. Several cells were seen in the area of hyperfluorescent plaque (Fig 4F). The orientation of cells and hyperfluorescent plaque was perpendicular to the nerve trunk. This is very different from orientation of cells in an intact (untransected) nerve (Fig 5A–5C). In contrast to the healing events in interconnected nerves, single point transections in dichotomously branching nerves resulted in degeneration of the segment distal to the laser ablation (Fig 6A and 6B).

### II. Two point transection of corneal nerves

**Fig 7:**
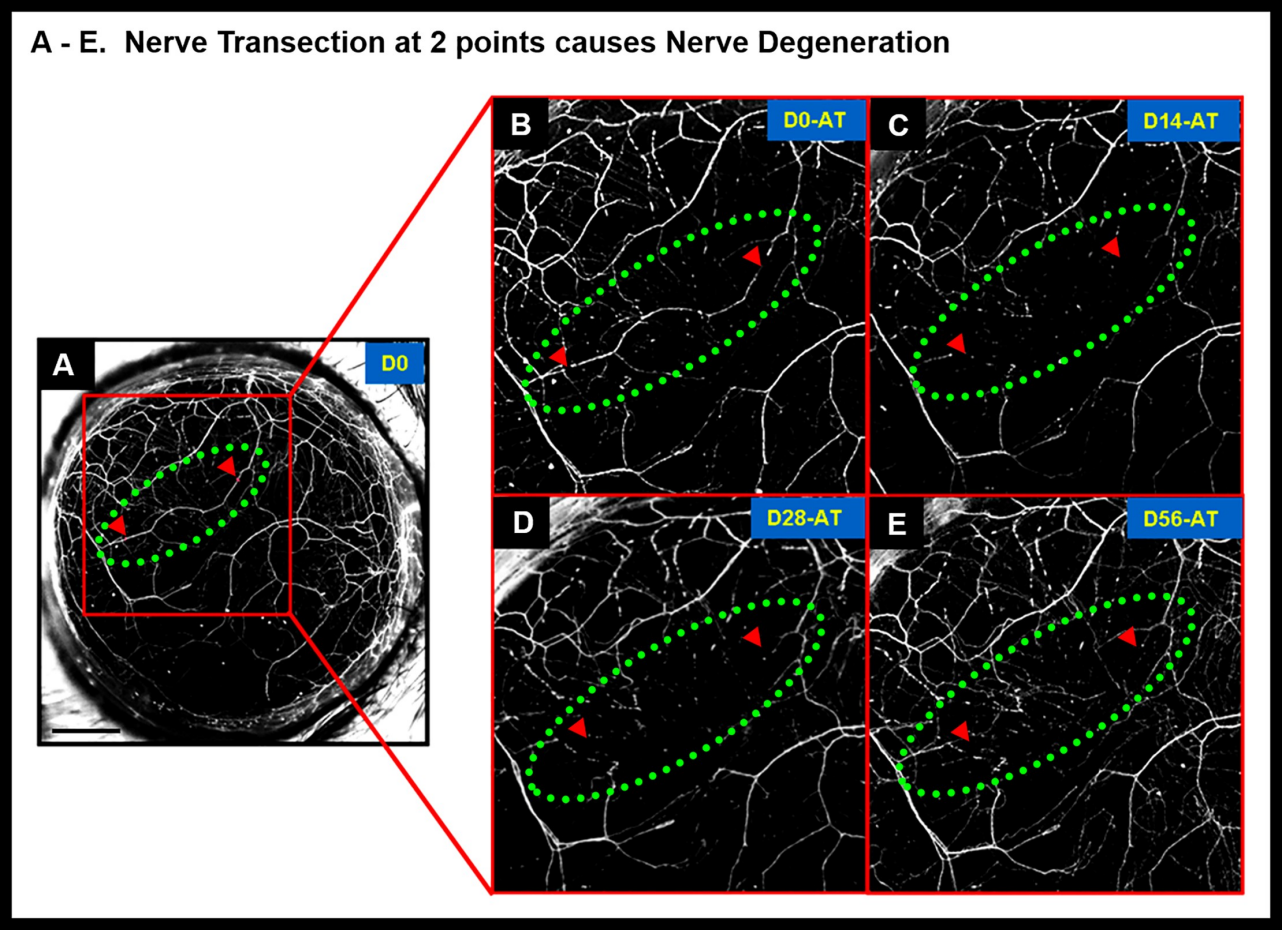
Nerve degeneration in interconnected corneal nerves after two point transection. Nerve transection at 2 points (red arrow heads indicate point of transection) caused loss of YFP fluorescence and disappearance of nerve segment that was enclosed within the cut ends. The red arrow heads depict position of the 2 cuts along the nerve segment (Panel A) and green dotted oval denotes the region of the cut nerve. Panels B, C, D and E are magnified images of the transected nerve at different time points (Days 0, 14, 28 and 56 respectively). In 2-point nerve transections, the nerve segment enclosed within the two laser cuts degenerated and did not result in nerve healing. Black scale bar in Panel A: 500 μm; AT refers to “after transection”.

Two-point transections were performed in interconnected nerves. Nerve segment degeneration was the more common response (n = 5/7 corneas) (Fig 7). Healing of the ablated area occurred infrequently (n = 2/7 corneas). During healing a hyperfluorescent plaque filled the gap created by the transection.

### III. Annular transection of corneal nerves

**Fig 8:**
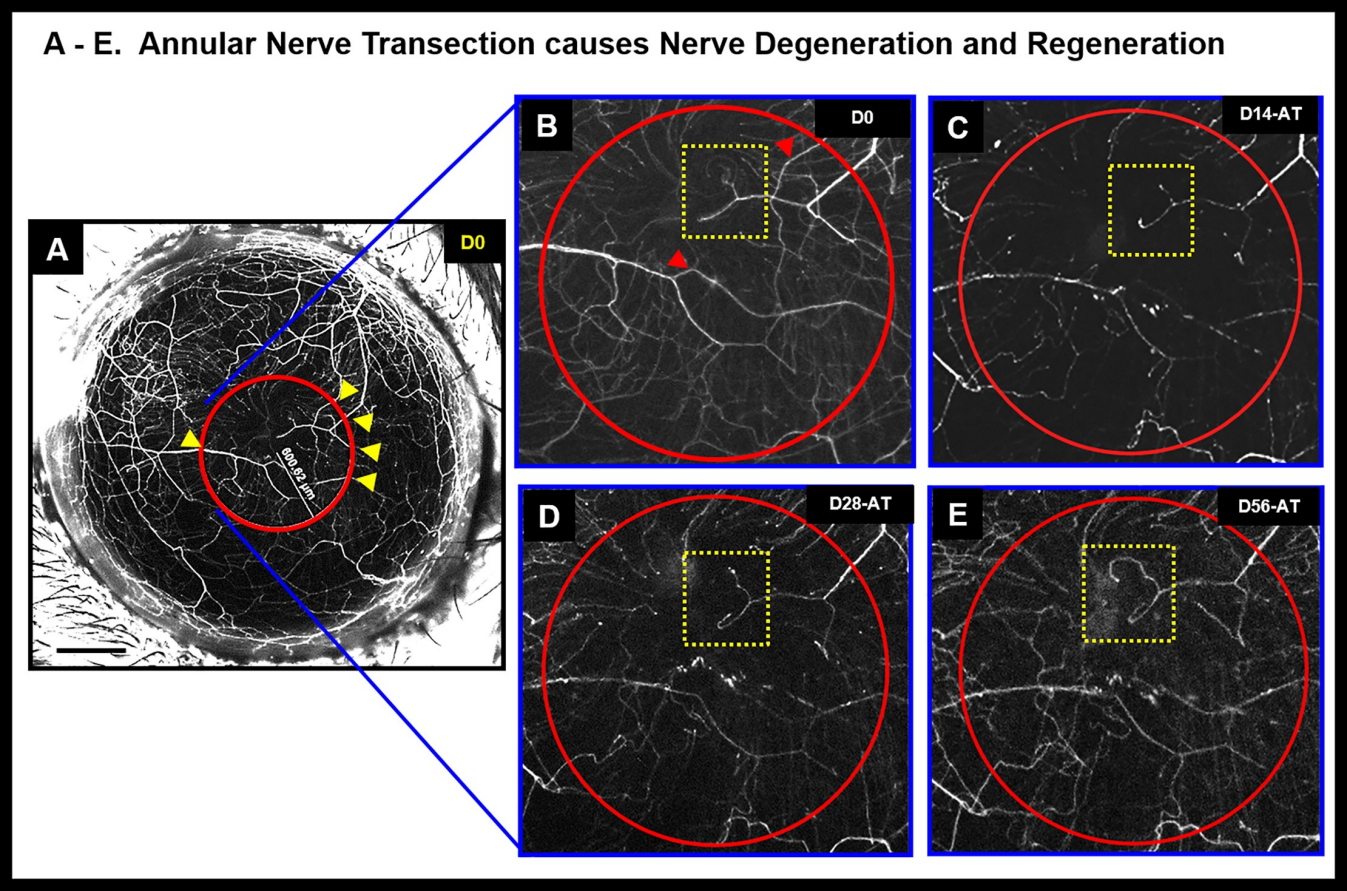
Annular nerve transection causes nerve degeneration and regeneration. Stereolumar image of annular nerve transection shows disappearance, reappearance or retention of nerve fluorescence. The yellow arrow heads depict positions of the nerve cuts within an area demarcated by the red circle. Panels B, C, D and E show magnified area within the red circle showing nerve changes corresponding to different time points after surgery (Days 0, 14, 28 and 56 respectively). The red arrow heads (Panel B) show nerve degeneration at different time points (Panels C–E). The yellow dotted area inset shows area of aberrant nerve regeneration; Black scale bar in Panel A: 500 μm; AT refers to “after transection”.

In annular nerve transections, single point cuts were made at nerves intersecting a concentric circular area (~ 600 μm radius). Annular transections caused some nerve trunks to degenerate, while others retained or regained fluorescence after an initial loss of fluorescence (Fig 8). In some areas aberrant nerve regeneration was seen characterized by regenerating nerve fronds.

## Discussion

Our study yielded the following findings: (1) Nerve patterns in corneas were found to be different in between mice including littermates; (2) Predominantly two nerve patterns occur in the cornea namely; (a) Interconnected nerve patterns with nerve trunks spanning one end of the limbus to the other and (b) Open-ended, dichotomous branching nerve patterns terminating at the corneal apex; (3) Nerve transection caused an initial zone of clearance at the point of injury and resulted in the formation of a hyperfluorescent plaque accompanied either by nerve healing or degeneration of nerve segment.

The key question we wanted to address in our current study was whether nerve regeneration would occur if there is no surgical plane present. The reason for asking this question is extremely relevant to disease conditions that specifically affect the nerves within the cornea, for example in diabetes or other immune/inflammatory etiologies of keratitis, where there are no surgical planes. In infectious and non-infectious keratitis, nerves may be damaged in the vicinity of the infiltrate in a manner similar to point transections performed in our experiments. We recognized that results from our prior investigations that surgically incised the cornea could be extrapolated to neurotrophic corneas that resulted from surgical interventions like lasik surgery, but those findings may not be extrapolatable to diseases of nerves that are not attributable to corneal surgery. Prior studies from our group [14, 15] involved the use of lamellar corneal flap surgery which yielded a wide-area transection of corneal nerves [14, 17]. Lamellar corneal flap surgery in our *thy1*-YFP mouse model caused an influx of inflammatory cells at the transected site at post-operative day 3 and subsequent aberrant nerve regenerative sprouting from the proximal stump of stromal trunks in a nerve pattern and density different from pre-operative levels. With in situ nerve transection we did not observe aberrant nerve regeneration after single point transections. Nerve trunks either healed (with a hyperfluorescent plaque at the site of transection) or degenerated. Limited amount of aberrant nerve regeneration was only observed in annular transection. Since published studies from our lab have already reported that “hairpin-like subbasal nerves” in the *thy1*-YFP mouse are prone to changes in nerve distribution pattern even in the absence of surgical intervention [14], all our nerve regeneration studies were carried out with reference only to “corneal stromal nerves”.

Nerve damage in the cornea is usually associated with common symptoms of ocular irritation, photophobia or pain without accompanying ocular surface disease or in patients with neurotrophic keratitis who may have no pain but significant ocular surface disease due to hypoesthesia [18–19]. Nerve damage due to surgical transections such as cataract surgery causes severe neuropathic pain [20]. Despite major developments and advances to ensure accuracy and precision in the field of corneal laser refractive surgery, procedures such as Laser *In Situ* Keratomileusis (LASIK), Photorefractive Keratectomy (PRK), Laser-Assisted Subepithelial Keratectomy (LASEK) and Epi-LASIK cause nerve damage and trigger wound healing and changes in keratocytes [21]. One of the major causes of post-refractive surgery Dry Eye Disease (DED) is due to sensory denervation caused by nerve transection during flap creation and excimer photoablation [22–23]. Erie have shown that subbasal nerve fiber density was 98% less than pre-operatively [24] and the ablation zone center showed complete absence of branched nerve fibers, 3 months post-surgery. Both Moilanen and Erie have demonstrated that subbasal nerve density was reduced by 87%, 75% and 60%, (at 3, 6 and 12 months respectively) after PRK, and returned to preoperative levels at 2 and 3 years postoperatively [24–25]. In another study using confocal microscopy, Erie’s team proved faster recovery of subbasal nerve density in the central cornea in PRK as compared to LASIK [26].

In other studies on BAK-induced neurotoxicity [16] we found that topical application of BAK to the mouse eye caused two forms of stromal nerve neurotoxicity; (i) reversible neurotoxicity (axonopathy) involving initial disappearance of nerve fluorescence and its subsequent reappearance in the same nerve pattern as before treatment and (ii) irreversible neurotoxicity (degeneration) characterized by complete loss of nerve fluorescence and gradual reappearance in a new pattern and location as compared to baseline. Our reversible neurotoxicity data was similar to that of Shriver and Dittel [27] which suggested that in *thy1*-YFP mice, loss of yellow fluorescence correlated with a disruption in axonal function and when inflammation was resolved and the mice recovered, a reversal of the axonal dysfunction was observed. However, in the case of irreversible neurotoxicity (nerve degeneration) wherein recovery was by regeneration, the density and pattern of the regenerated nerves differed greatly from normal innervation, and functional characteristics were unknown.

In conclusion, we performed *in situ* transection of mouse corneal stromal nerves using a far-infrared XYRCOS Laser, which permitted non-contact transection of corneal nerves. We observed evidence of nerve healing. Further studies need to be carried out in order to evaluate and characterize the functional significance of nerve healing after *in situ* nerve ablation.

## Supporting information

S1 File Materials and methods for corneal Brightfield imaging and fluorescein staining after laser nerve transection using *in vivo* stereofluorescent microscopy. (DOCX)

S1 Fig Corneal Brightfield imaging and fluorescein staining after laser nerve transection. On day 0 before nerve transection, in addition to stereolumar imaging of corneal nerves (A, B), Bright-field image (C) and Fluorescein staining (D) images were taken. Stereofluorescent images were also taken after nerve transection on day 0 (point of transection denoted by a red dot in panel E and red arrow in panels B and F). Bright-field image (G) and fluorescein staining (H) showed absence of superficial punctate keratitis confirming absence of epithelial cell injury. At Day 3, clear, transparent cornea (I) and absence of superficial punctate keratitis (J) confirmed absence of epithelial cell injury after nerve transection; D0 = Day 0; D3 = Day 3.
(TIF)
