# Supplementary material for: Corneal nerve healing after in situ laser nerve transection
Source: PLoS One. 2019 Jun 27;14(6):e0218879. doi: 10.1371/journal.pone.0218879 (PMC6597159; doi:10.1371/journal.pone.0218879)
Supplement: S1 File — (DOCX) [file pone.0218879.s001.docx]

**Supporting Information**

**Materials and Methods**

**Ethics Statement**

All animal experiments were conducted in strict accordance with the recommendations in the Guide for the Care and Use of Laboratory Animals of the National Institutes of Health. The animal protocol was approved by the Institutional Animal Care and Use Committee (IACUC) of the University of Illinois at Chicago (Protocol Number: 13-159). *Thy1*-YFP neurofluorescent homozygous adult mice (6–8 weeks old) were purchased from Jackson Laboratories (Bar Harbor, ME), and colonies were established by inbreeding. For *in vivo* experiments, mice were anesthetized with intraperitoneal injections of ketamine (20 mg/kg; Phoenix Scientific, St. Joseph, MO) and xylazine (6 mg/kg; Phoenix Scientific). For terminal experiments, mice were sacrificed according to the IACUC protocol. Euthanasia was performed by CO_2_ inhalation followed by cervical dislocation in adult animals. These procedures were chosen based on their reproducibility and the fact they cause no discomfort to the animals. These methods are consistent with the recommendations of the Panel on Euthanasia of the American Veterinary Medical Association. All efforts were made to minimize suffering.

***In Situ* Laser Transection**

Experiments involving *in situ* transection of the mouse corneal stromal nerves were performed using a far-infrared XYRCOS Laser (Hamilton Thorne Inc; Beverly, MA) which permits non-contact ablation of targeted membranes or structures. The XYRCOS laser module consists of a high power, Class 1, 1460 nm infrared laser plus RED-i target locator integrated into a 20X objective and is compatible with most inverted microscopes. The XYRCOS laser attaches to the turret just like a typical objective and allows full use of all the microscopes standard features, such as fluorescence and Hoffman imaging. In addition, the laser is factory-aligned and locked in place to ensure safe ablation. In our setup the XYRCOS Laser was attached to a Zeiss AxioExaminer A1 Upright Microscope (Carl Zeiss Microscopy, Thornwood, NY). After initial baseline images (Day 0) before surgery using a Zeiss Stereolumar microscope (details in next section), nerve transection was performed using the XYRCOS laser (Pulse: 200 µs; Power: 100%); single nerve cut in either an interconnected trunk or stromal nerve ending. The mice were followed up after surgery and sequential stereomicroscopic images were taken on Day 3 after nerve transections to assess changes in fluorescence intensity and pattern of regenerating nerve fronds, if any. Only stromal nerves were included in the analysis. Subbasal hairpin nerves were excluded.

***In Vivo* Stereofluorescent Microscopy**

Initial baseline (Day 0) and serial imaging after nerve transection surgeries was performed using a fluorescence stereomicroscope (StereoLumar V.12, Carl Zeiss Microscopy, Thornwood, NY) equipped with a digital camera (Axiocam MRm) and software (AxioVision 4.0) as described previously [14]. An anesthetized mouse was placed on the stereoscope stage. Seven microliters of proparacaine (0.5%, Bausch & Lomb, Tampa, FL) was applied for 3 min, and the pupil was constricted with 0.01% Carbachol intraocular solution (Miostat, Alcon) for 5 min. Z-stack images were obtained at 5-μm intervals and compacted into one maximum intensity projection (MIP) image after alignment using Zeiss AxioVision software. Brightfield images and corneal fluorescein staining images were taken (S1 Fig) before and after nerve transection on Day 0 and on Day 3 after transection to confirm corneal transparency after nerve transection surgeries.

**Corneal Fluorescein Staining**

Corneal ﬂuorescein staining was performed by applying 0.5 lL of 0.1% ﬂuorescein by micropipette into the inferior conjunctival sac of the eye as reported previously^1^. The cornea was imaged using the stereoﬂuorescent microscope using blue ﬁlter 3 minutes after ﬂuorescein instillation. The images were analyzed with a standardized (National Eye Institute) grading system^2^.

**Figure and Figure legend**

**S1_Figure**

**S1_Fig: Corneal Brightfield Imaging and Fluorescein Staining after laser nerve transection.** On day 0 before nerve transection, in addition to stereolumar imaging of corneal nerves (A, B), Bright-field image (C) and Fluorescein staining (D) images were taken. Stereofluorescent images were also taken after nerve transection on day 0 (point of transection denoted by a red dot in panel E and red arrow in panels B and F). Bright-field image (G) and fluorescein staining (H) showed absence of superficial punctate keratitis confirming absence of epithelial cell injury. At Day 3, clear, transparent cornea (I) and absence of superficial punctate keratitis (J) confirmed absence of epithelial cell injury after nerve transection; D0=Day 0; D3=Day 3.

**References:**

1. Lin Z, Liu X, Zhou T, et al. A mouse dry eye model induced by topical administration of benzalkonium chloride. Mol Vis. 2011;Jan 25;17:257–264.

2. Lemp MA. Report of the National Eye Institute/Industry workshop on clinical trials in dry eyes. CLAO J. 1995;21: 221–232.
