# Supplementary figures and images for: Corneal nerve healing after in situ laser nerve transection
Source: PLoS One. 2019 Jun 27;14(6):e0218879. doi: 10.1371/journal.pone.0218879 (PMC6597159; doi:10.1371/journal.pone.0218879)

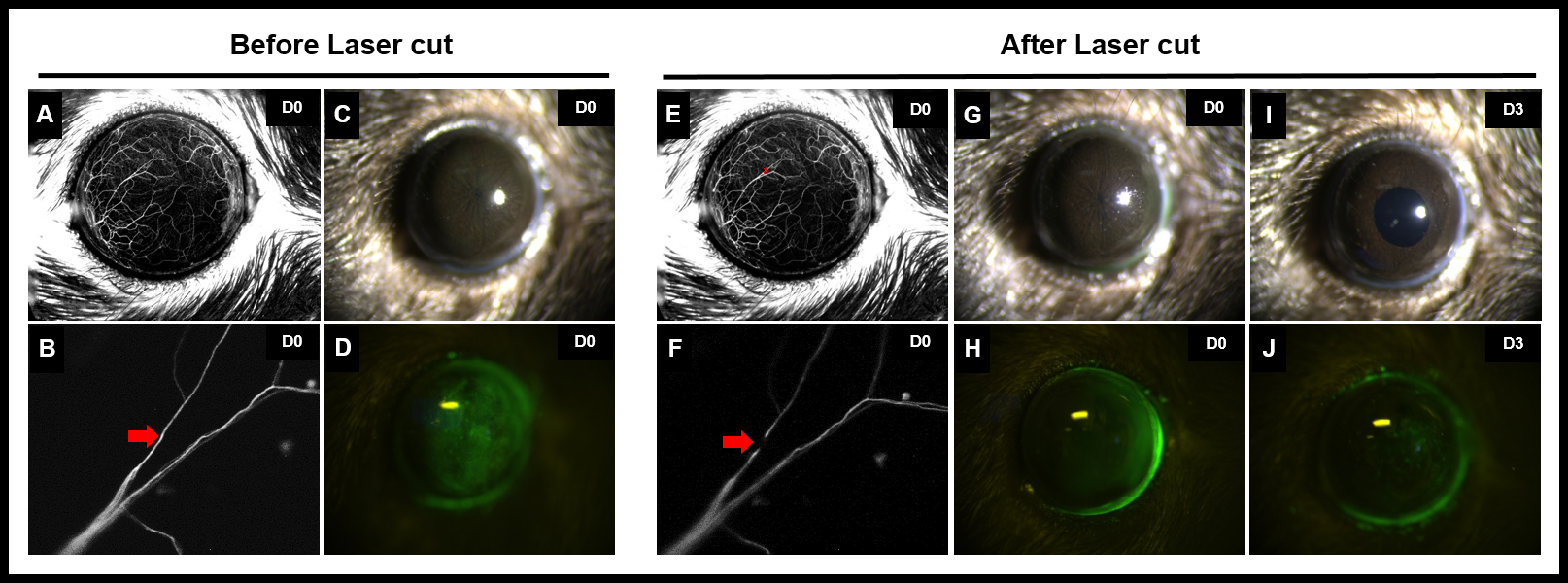

Supplement: S1 Fig — On day 0 before nerve transection, in addition to stereolumar imaging of corneal nerves (A, B), Bright-field image (C) and Fluorescein staining (D) images were taken. Stereofluorescent images were also taken after nerve transection on day 0 (point of transection denoted by a red dot in panel E and red arrow in panels B and F). Bright-field image (G) and fluorescein staining (H) showed absence of superficial punctate keratitis confirming absence of epithelial cell injury. At Day 3, clear, transparent cornea (I) and absence of superficial punctate keratitis (J) confirmed absence of epithelial cell injury after nerve transection; D0 = Day 0; D3 = Day 3. (TIF) [file pone.0218879.s002.tif]
